# Efficacy of trypan blue in posterior capsulorhexis with optic capture in pediatric cataracts [ISRCTN48221688]

**DOI:** 10.1186/1471-2415-6-12

**Published:** 2006-03-16

**Authors:** Namrata Sharma, Ramamurthy Balasubramanya, Vijay K Dada, Rasik B Vajpayee

**Affiliations:** 1 Rajendra Prasad Centre for Ophthalmic Sciences, All India Institute of Medical Sciences, New Delhi, INDIA

## Abstract

**Background:**

To evaluate the efficacy of trypan blue (0.06%) in posterior capsulorhexis with optic capture in pediatric cataracts.

**Methods:**

In this prospective randomized controlled study, trypan blue dye assisted posterior capsulorhexis with optic capture was performed in 18 eyes (group 1) and no dye was used for posterior capsulorhexis (group 2) in 17 eyes.

**Results:**

The mean size of the posterior capsulorhexis was 4.6 +/-1.77 mm and 4.0 +/- 0.93 mm in the group 1 and 2 respectively. Optic capture was possible in 17 eyes in the group 1 and 11 eyes in the group 2.

**Conclusion:**

Trypan blue facilitates posterior capsulorhexis with optic capture of AcrySof IOL in cases of pediatric cataracts.

## Background

Maintenance of a clear visual axis after congenital cataract surgery is mandatory in order to obtain an optimal visual outcome. Proper management of the posterior capsule and early rehabilitation with amblyopia therapy are major factors, which decide the visual outcome of pediatric cataract surgery. The risk of posterior capsule opacification in children is almost 100% and prevents visual rehabilitation[1]. Although Nd:YAG capsulotomy is a relatively safe procedure, its use is limited to older cooperative children[2]. There is an increasing tendency towards intraocular lens (IOL) implantation in children, which requires at least a part of posterior capsule to be preserved. An anterior vitrectomy may be necessary to maintain clear visual axis even after performing primary posterior curvilinear capsulorhexis (PCCC) [3] as the anterior hyaloid acts as scaffold for lens epithelial migration and regenration[4].

Various therapeutic options which have been attempted to maintain a clear visual axis include lensectomy with anterior vitrectomy and primary posterior capsulorhexis with optic capture of the IOL with or without anterior vitrectomy[5,6]. Gimbel and DeBroff proposed capturing a polymethylmethacrylate optic through primary posterior capsulorhexis to keep the visual axis clear[7]. Hydrophobic acrylic intraocular lens (IOL) may be captured through a posterior capsulorhexis and combines the advantages of optic capture with a smaller incision with decreased inflammatory response[8]. Performing posterior capsulorhexis with optic capture requires more surgical expertise[9] because of thinner and more fragile posterior capsule, which makes it more difficult to visualize. Although, trypan blue has been successfully used for anterior capsule staining in white cataracts[10,11], and in cataracts with corneal opacities[12]. Its efficacy has also been reported in the staining of posterior plaque removal in pediatric cataracts[13] but to the best of our knowledge there are very few reports[14,15] of trypan blue dye assisted PCCC with optic capture in pediatric cataracts.

In this study, we evaluated the efficacy of trypan blue staining of the posterior capsule for the performance of PCCC with optic capture of hydrophobic acrylic IOL in pediatric cataracts.

## Methods

This prospective randomized controlled study, thirty five eyes of 26 children, with age ranging from 5 years to 12 years with congenital or developmental cataract, who underwent lens aspiration with three piece AcrySof MA60BM (Alcon, Fort Worth, TX) (hydrophobic acrylic IOL) IOL implantation and optic capture from January 2002 to December 2002 were analyzed. In-group 1 (18 eyes); trypan blue dye assisted PCCC was done and in-group 2 (17 eyes); no dye was used. Informed consent was taken from the parents. Consecutive cases with visually significant lenticular opacity (3 mm or larger) were included. Eyes with traumatic cataract and associated ocular abnormalities were excluded.

The IOL power was prescribed as per the standard protocol followed in our centre. Emmetropic power was determined using the SRK II formula and the power of IOL to be inserted was calculated by under correcting by 10% in children between 2 and 8 years and emmetropic power in those above 8 years of age[16]. In those cases, which had refractive errors and unilateral cataract, measurements in the fellow-eye were taken into consideration for determining IOL power. In very young and uncooperative children, axial length measurement and keratometry with the hand held automated keratometer was performed just prior to performing cataract surgery under general anesthesia.

The pupil was dilated with atropine 1% ointment instilled three times a day two days prior to the day before surgery. Homatropine eye drops 2 % were instilled four times, thirty minutes prior to surgery. All surgeries were performed under general anesthesia. A clear corneal incision 3.2 mm was made with the keratome at least 2.0 mm inside clear cornea. Sodium hyaluronate 1.4% (Healon GV, Advanced Medical Optics, Santa Ana, CA.) was injected and an anterior capsulorhexis was performed using a capsulorhexis forceps. After hydrodissection, the lens material was aspirated using an automated irrigation aspiration port or a bimanual irrigation/aspiration (I/A).

In order to stain the posterior capsule an air bubble was injected and trypan blue (Shah & Shah, Calcutta, India) 0.1 ml, 0.06 % was instilled under the air bubble. The dye was then washed with the balanced salt solution. Sodium hyaluronate 1.4% (Healon GV, Advanced Medical Optics, Santa Ana, CA.) was then instilled to inflate the capsular bag. A small nick was made on the posterior capsule and sodium hyaluronate 1.4% (Healon GV, Advanced Medical Optics, Santa Ana, CA.) was instilled below this nick to push the anterior vitreous face backwards. The stained edge of the capsule was then lifted and grasped with the capsulorhexis forceps and the posterior capsulorhexis was performed. Bimanual anterior vitrectomy was performed only in those cases in which vitreous prolapsed through the PCCC.

**Figure 1:**
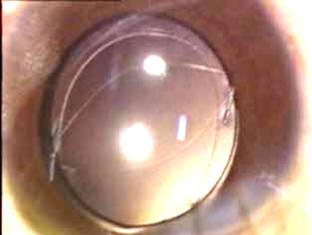
Photograph showing three piece AcrySof MA60BM (Alcon, Fort Worth, TX) optic capture through trypan blue assisted posterior capsulorhexis opening.

A foldable three piece AcrySof MA60BM (Alcon, Fort Worth, TX) (hydrophobic acrylic IOL) was then implanted in the bag and two sinskey hooks were used to capture the lens in the posterior capsulorhexis. In cases in which the PCCC was capturable i.e. optimum size and shape, an optic capture was done (Figure 1). The incision was sutured using 10-0 monofilament nylon and the edges of the wound of the main tunnel as well as the side ports were hydrated. In the group 2, all steps were similar except for the staining of the posterior capsule.

Postoperative topical treatment comprised of dexamethasone/neomycin eyedrops 6 times a day tapered over 2 weeks, diclofenac sodium eyedrops 3 times a day for 1 month, and tropicamide 1% eyedrops three times a day for 2 weeks. All patients were examined on the first postoperative day and then at regular intervals (i.e. 1 week, 2 weeks, 1 month and 3 months. At each visit, best corrected visual acuity, visual axis clarity and IOL related problems were noted.

## Results

The mean age of the patients in the group 1 and 2 were 8.3 ± 2.1 years and 7.9 ± 2.4 years. The mean size of the anterior capsulorhexis in group 1 and 2 was 5.9 ± 1.74 mm and 5.8 ± 0.92 mm respectively (p = 0.072). The mean size of the posterior capsulorhexis was 4.6 ± 1.77 mm and 4.0 ± 0.93 mm in the group 1 and 2 respectively (p = 0.04) (Table 1).

**Table 1:** Comparison of group 1 (trypan blue assisted PCCC) and group 2 (no dye was used).

	***No. of eyes***	***Age (mean ± SD)***	***Size of ACCC (mean ± SD)***	***Size of PCCC (mean ± SD)***	***optic capture of IOL in PCCC opening***	***Anterior vitrectomy***
**Group 1**	18 eyes	8.3 ± 2.1 yrs	5.9 ± 1.74 mm	4.6 ± 1.77 mm	17 eyes	2 eyes
**Group 2**	17 eyes	7.9 ± 2.4 yrs	5.8 ± 0.92 mm	4.0 ± 0.93 mm	11 eyes	2 eyes

ACCC – anterior continuous curvilinear capsulorhexis, PCCC – posterior continuous curvilinear capsulorhexis, IOL – Intraocular lens

**Figure 2:**
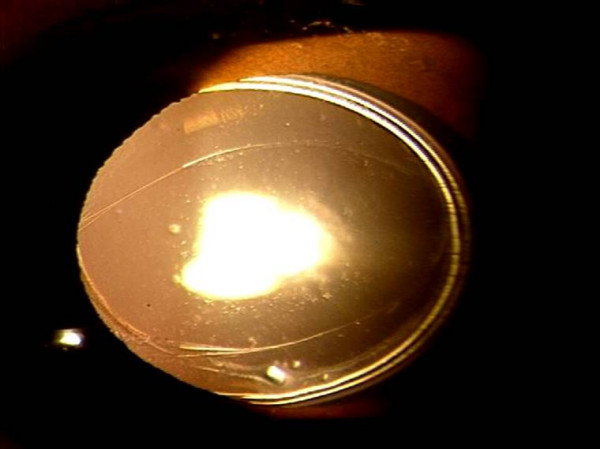
Photograph showing three piece AcrySof MA60BM (Alcon, Fort Worth, TX) optic capture through posterior capsulorhexis opening in which trypan blue was not used.

Subjectively, the posterior capsule staining aided in the visualization of the posterior capsule and the stained edge was better visualized as compared to the non-stained capsule. Anterior vitrectomy was required in two eyes each in the two groups. Optic capture was possible in 17 eyes in the group 1 and 11 eyes in the group 2 (figure 2). In the group 1, the posterior capsulorhexis was not capturable as the capsule was fibrosed. In the group 2, the posterior capsulorhexis was not capturable as it was small in two eyes, eccentric in two eyes and irregular in two eyes.

## Discussion

PCCC is a posterior continuous curvilinear capsulotomy technique described by Gimbel[7]. PCCC is also done for converting an irregular rupture of the posterior capsule into a circumscribed cut not extending to the equator[17,18]. It can also be used for the removal of posterior capsular plaques in posterior subcapsular or polar cataracts[19].

Recently, the use of PCCC combined with optic capture of an IOL[7] and/or anterior vitrectomy[20] successfully evolved for delaying the development of posterior capsule opacification (PCO) or secondary membrane formation in pediatric cases. Primary posterior capsulotomy, in the form of PCCC, is especially important in younger children for maintaining a long-term clear visual axis in order to prevent the development of amblyopia[9].

Anterior capsule staining to facilitate anterior capsulorhexis is a well-established technique. Posterior capsulorhexis is technically more difficult to visualize, as it is thinner, more fragile and more elastic as compared to the anterior capsule. Frequent re-grasping and re-lifting of the capsular edge is required to facilitate the posterior capsulorhexis. Attempting PCCC in the presence of poor visibility associated with positive vitreous pressure is difficult and may cause an inadvertent radial tear extending toward the equator.

It is also more critical to achieve a posterior capsulorhexis especially in cases where optic capture is planned and a 'capturable posterior capsulorhexis' is mandatory. One of the most important factors for a "capturable" posterior capsulorhexis is the size of "IOL optic". While it is possible to capture the IOL with a optic diameter of 5.0 mm to 5.5 mm through a posterior capsulorhexis of approximately 3.75 mm to 4.0 mm. On the other hand, the IOL with 6 mm optic diameter may necessitate slightly larger (about 4.25 mm to 4.5 mm) posterior capsulorhexis for a successful IOL optic capture, without excessive ovalization of the posterior capsulorhexis opening. The staining of the posterior capsule with the trypan blue results in better visualization of the blue edge of the capsule beneath an underlying red reflex. The highlighted blue edge can then be easily re-grasped, re-lifted and re-oriented in a desired direction so as to achieve a circular and optimally sized posterior capsulorhexis.

In one case a posterior plaque was present and in another eye posterior capsular fibrosis was present which were delineated very well with the trypan blue dye. The morphology of the posterior capsule was discerned clearly so that a 'capturable' posterior capsulorhexis was facilitated.

Further, the spindle of the posterior capsule, which was the hallmark of achieving the capture of the posterior capsulorhexis, was better visualized. Thus the stained edge of the posterior capsulorhexis was manipulated more easily in the cases where trypan blue was used as compared to the cases in which no dye was used. The vitreous staining was not seen in any cases in the current series. However, possibility of inadvertent vitreous staining especially in a case of traumatic cataract with compromised zonular apparatus should be kept in mind[21].

The sizes of the anterior and the posterior capsulorhexis were comparable in the two groups. This may be attributed to the expertise and the surgical skills of the surgeons as experienced surgeons did the surgeries in both the groups. The difference in the two groups that is the stained and the non-stained group may become more significant if larger number of cases is taken. Further, trypan blue staining of the posterior capsule may be especially useful in congenital cataract surgeries, which are being undertaken by the novice surgeons or the surgeons who are in their learning curve.

As trypan blue is a potentially carcinogenic vital dye and its possible long-term side effects are unknown, the lowest effective concentration should be used particularly in childern. A concentration lower than 0.1% was effective in staining the anterior capsule even under dispersive viscoelastic material [22]. In our study none of our IOL were stained with trypan blue.

## Conclusion

Trypan blue even in the lower concentration (0.06%) facilitates appropriate staining of the posterior capsule for performing PCCC in pediatric cataracts. When trypan blue was used PCCC was complete in 94.4% compared to 64.7% when it was not used. We recommend that studies should be undertaken in larger number of subjects and results of achieving a posterior capsulorhexis and optic capture may be compared with and without trypan blue staining especially in the hands of novice surgeons.

## Competing interests

The author(s) declare that they have no competing interests.

## Authors' contributions

All authors contributed to designing, implementing and analyzing the study.

## Pre-publication history

The pre-publication history for this paper can be accessed here:


